# The Wants of the Profession

**Published:** 1862-03

**Authors:** J. Taft


DENTAL REGISTER OF THE WEST.

VOL. XVI.] MARCH, 1862. [No. 3.

### Original Essays and Communications.

### THE WANTS OF THE DENTAL PROFESSION.

BY J. TAFT.

Read before the Indiana State Dental Association, at Lafayette, Jan. 14th, 1862.

UNDER this caption I present for your consideration some of those things which seem to be, and we think are, required for the further development of dental *science* and *art*.

It may seem strange to speak of a *more* rapid development, or an increase of the means and instrumentalities for that purpose, of a profession, which has in a few brief years leaped into existence, and is now coursing a brilliant career, unparalleled by any other profession; having aggregated to itself all the ordinary agencies for the development of human progress. Notwithstanding all this, there is much that can, and should, aye must be done for further development; there is much that is yet in a very imperfect condition; much that should be superceeded by different and better modes; false notions to be displaced, and correct ones to be substituted; ignorance to be eradicated by the introduction of true knowledge; prejudices to be overcome, and correct principles to be implanted in their stead.

It is true, we have the ordinary enginery for professional progress and attainments in operation; these are not, however, in the best working condition. It would perhaps be too much to expect that these should not be effected by the storm of revolution which sweeps over all things else, The professions should be, and perhaps are, less affected by such things than any other department of human occupation. Every one is familiar with the fact, in his own experience, that in the practice of the dental profession, there are many things about which he may say, *it is imperfect.* Who will venture the assertion, that anything we do is perfect; with emphasis may the question be asked, in reference even to our best operations. With joy and gladness would we hail him who with justice could exclaim, with the ancient sage, "*Eureka.*" None, even those of the highest attainments in the profession will venture to set up such a claim; desire is not satisfied—content is not obtained, rest is not found. We all see points and particulars, which we desire were otherwise, and which we would most gladly improve or perfect. But alas, our attainments are insufficient. There are men, however, in the dental profession, as in every department of human action, who seem to lay the flattering unction to their souls, that whatever they do is perfect. For such "'twould be folly to be wise," for it would immediately spoil the value which they place upon their own efforts; they are blessed with content, and self-congratulation through ignorance, which is too often riveted to them with prejudice.

In considering the wants existing in the practice of the dental profession, we shall refer to those above, and beyond the ordinary full measure of attainments.

It is quite probable that some of these requirements will never be answered; we have, however, a grand earnest in the past for the future. We need only refer to two or three things in verification of this. Within the last ten years the method of filling teeth has been completely revolutionized; and the method of treatment, of some diseases, to which the teeth are subject, has been almost entirely originated; three or four different methods of mounting artificial teeth—and each of them good in many respects—have been originated within the same time. But notwithstanding the progress made, in these and other particulars; there is much yet wanting in every one of them. I propose to enumerate some of these, and then briefly endeavor to present the proposition, that scientific attainments constitute the only basis upon which real progress and true practice can be predicated.

In regard to deficiencies in practice, they may be divided into those pertaining to operations, and those pertaining to remedial and hygenic treatment. In regard to operations, two sources of difficulty exist, one in regard to the character of materials employed, and the other to the manner in which they are used. Of the material used in dental practice, we all, doubtless, have more or less familiarity. We know their merits and demerits. These may be subdivided into those which are inherent, and those which are accidental; the former having reference to the presence of some objectionable quality, or the absence of some desirable condition.

In regard to the materials employed by the dentist in practice, there are none perfect; some have objectionable properties, and some are made so by the mode of preparation some require a great amount of labor and manipulation to make them efficient in the accomplishment of the object for which they are designed.

Gold as a material for filling teeth, has many valuable qualities, but it has two or three peculiarites that render it disadvantageous; its color is objectionable, especially for filling cavities exposed to view, in the anterior teeth; the contrast between it and the clear dentine or enamel, is too great for beauty, or a correct taste.

The conducting power of the metal is perhaps its most objectionable quality as a material for filling the teeth. In sensitive teeth, this property of gold, is oftentimes a source of great inconvenience, and sometimes occasions serious results; in many cases by the changes of temperature, thus brought to bear upon susceptible dentine, a high state of exalted sensibility is induced, so much so as to be exceedingly annoying to the patient, and in some instances to destroy the vitality altogether. It is because gold does not in this respect afford a competent protection to the exposed dentine, that the demand for agents to destroy or prevent exalted sensibility of the dentine, at the time of filling, is so great. You are perhaps *all* aware of the fact, that if a cavity of decay in a tooth, though exceedingly sensitive, be filled with a non-conducting substance, and one that will shield it from other injurious influences, for example, "Hill's Stopping," the part will in a very short time, return to its normal condition, the exalted sensibility be entirely removed.

All metallic fillings in proportion to their power of conducing heat, are so far objectionable as a material for filling teeth.

Other substances better in this respect than metals, are objectionable, in other particulars, as for instance, their liability to be destroyed, etc. Will science ever enable us to overcome this objection in the metals, or shall we search for some other material that is better, as a whole, than those we now have.

The accidental defects in materials employed by the dentist, are those that occur by the mode of preparation. Some natural defects may be removed by skillful preparation and modification; and in the absence of these, the most obvious defects may remain, sufficient to thwart the efforts of the most skillful manipulator in the use. The defects which now particularly claim our attention, are those which remain after the present highest skill has been employed in the preparation.

The greatest deficiencies are those which reach the patient, those that can be removed previous to this, though it may be at the cost of much labor, are certainly of minor importance.

The great demand made upon time, skill and labor, for the efficient introduction of gold into the cavities of teeth, constitute another objection to its use. It is often said that it is a matter of little consequence, how much time or labor is expended in filling a tooth, if it is done well, and accomplishes the desired objects.

Perhaps this allegation is made, more because these are absolutely necessary in the production of a good gold filling, than because the time and labor is of little consequence.

If a material can be produced, with which half a dozen efficient fillings could be made, in the time ordinarily required for one gold filling; a great advance certainly would have been made, several points would be gained. The fees for filling, could be very much reduced, and the operator be as well remunerated, and thus this valuable operation would be brought within the reach of thousands, who now regard it as altogether too expensive for their circumstances. By this means, a far greater range of usefulness would be opened up to the profession, than exists at present.

In filling teeth with gold, our profession has always stood in a dilemma.

If we reduce the time and labor of the operation, in order to accommodate the endurance of the patient, and bring the fee within range of his limited ability, then the work is inefficient.

If we determine to make every step in every operation perfect, then we draw largely upon our own time, skill and labor, and heavily upon the patience, endurance, and money too, of the patient. Thus we see it is desirable to have something better than gold filling for teeth.

In regard to substitutes for the natural teeth, the rapid changes through which this kind of work has passed, fully testify that the ultimatum has not been reached, though it is certainly being approximated; facility, rapidity of production, and perfection, as well as cheapness, is always desirable when there is great demand.

More progress is being made in all these respects, in this department, than for the preservation of the natural teeth. Every day brings us improvements in artificial teeth—in their beauty and strength. Changes and improvements are constantly being made in the construction of artificial dentures, both in the style and manner of accomplishing it. There is a continual outreaching for improvement, in the material for mounting teeth.

In the instruments and appliances used by the dentist, in the practice of his profession, there is yet much to be accomplished before perfection is attained. The mention of this fact is quite sufficient to bring it impressively to the mind of every one. The few things already enumerated, have special reference to the practice of the profession. There are other particulars, in which equally as great wants exist; I only stop now to mention two or three. And, first, a want of appreciation of the attainments requisite to constitute a dentist, in the full meaning of the term. How few, aye, are there any, who possess all the knowledge, that can be turned to good account in practice; verily there is not one. And with many of us, how little do we know, compared with what our consciousness tells we should know; with many of us an increase of knowledge seems only to give more enlarged views of our ignorance; instead of raising us out of the mire, only enables us to measure its depths a little more accurately. With many of our brethren, their idea of professional attainments, is exceedingly circumscribed; embracing only a knowledge of the manner of performing a certain routine of manipulation; with this, resting satisfied—not even making an effort to ascertain the circumstances, either present or future, that may influence their operations.

There is in the profession a failure to recognize the value and importance of good dental operations.

This want of appreciation of the importance and value of good dental operations, also exists to a lamentable extent, among the people. The strongest evidence of this, we have in the fact, that so many are willing to accept imperfect and inefficient operations. Here lies a fundamental difficulty. If the people demanded better dentists, and better operations, we would have them.

We only design further to present the proposition that science is the only true basis upon which to predicate correct practice.

This proposition, like some others, upon the mere statement of it, seems almost self-evident. A man may, in a routine way, accomplish many things, possessing at the time, but little more knowledge than that of the mere acts which he performs, together with those in the immediate connection; this he may do when the modifying circumstances are few and simple; but when it is otherwise, he can accomplish nothing with certainty of desired result. A familiarity with principles and laws, will enable those engaged in any department of human industry, to call to their aid instrumentalities that the ignorant know not of, and can not approach.

In some men we find examples of plus science, and minus practice—such are rarely useful, except to aid those who execute; others are all practice, and no science, such rarely produce results of great merit.

There are occasionally persons with a high order of natural endowments, with little cultivation, they often originate that which is valuable, though perhaps more by accident than by design. He only, who has the most extensive and intimate acquaintance, with the agencies he may employ, is the man of power and efficiency.

### THE DECIDUOUS TEETH.

BY J. TAFT.

